# P53-regulated miR-320a targets PDL1 and is downregulated in malignant mesothelioma

**DOI:** 10.1038/s41419-020-02940-w

**Published:** 2020-09-14

**Authors:** Caterina Costa, Paola Indovina, Eliseo Mattioli, Iris Maria Forte, Carmelina Antonella Iannuzzi, Luca Luzzi, Cristiana Bellan, Simona De Summa, Enrico Bucci, Domenico Di Marzo, Marisa De Feo, Luciano Mutti, Francesca Pentimalli, Antonio Giordano

**Affiliations:** 1 Cell Biology and Biotherapy Unit, Istituto Nazionale Tumori-IRCCS-Fondazione G. Pascale, I-80131 Napoli, Italy; 2 Sbarro Institute for Cancer Research and Molecular Medicine, Center for Biotechnology, College of Science and Technology, Temple University, Philadelphia, PA 19122 USA; 3 Institute for High Performance Computing and Networking, National Research Council of Italy (ICAR-CNR), Naples, Italy; 4 Histopathological Unit, IRCCS-Istituto Tumori “Giovanni Paolo II”, Viale Orazio Flacco 65, 70124 Bari, Italy; 5 Thoracic Surgery Unit, Department of Medicine, Surgery and Neuro Sciences, Diagnostic Imaging, University of Siena, Azienda Ospedaliera Universitaria Senese, Siena, Italy; 6 Department of Medical Biotechnologies, University of Siena, Siena, 53100 Italy; 7 Molecular Diagnostics and Pharmacogenetics Unit-IRCCS-Istituto Tumori “Giovanni Paolo II”, Viale Orazio Flacco 65, 70124 Bari, Italy; 8 Department of Cardiothoracic Sciences, Università degli Studi della Campania ‘L. Vanvitelli’ c/o Monaldi Hospital, Via L. Bianchi, 80131 Napoli, Italy

**Keywords:** Mesothelioma, Oncogenesis

## Abstract

Malignant pleural mesothelioma (MPM) is an aggressive cancer, related to asbestos exposure, which has a dismal prognosis. MPM diagnosis is late and often challenging, suggesting the need to identify more reliable molecular biomarkers. Here, we set out to identify differentially expressed miRNAs in epithelioid, biphasic, and sarcomatoid MPMs versus normal mesothelium and explored specific miRNA contribution to mesothelial tumorigenesis. We screened an LNA™-based miRNA-microrray with 14 formalin-fixed paraffin-embedded (FFPE) MPMs and 6 normal controls. Through real-time qRT-PCR we extended the analysis of a miRNA subset and further investigated miR-320a role through state-of-the-art techniques. We identified 16 upregulated and 32 downregulated miRNAs in MPMs versus normal tissue, including the previously identified potential biomarkers miR-21, miR-126, miR-143, miR-145. We showed in an extended series that miR-145, miR-10b, and miR-320a levels can discriminate tumor versus controls with high specificity and sensitivity. We focused on miR-320a because other family members were found downregulated in MPMs. However, stable miR-320a ectopic expression induced higher proliferation and migration ability, whereas miR-320a silencing reduced these processes, not supporting a classic tumor-suppressor role in MPM cell lines. Among putative targets, we found that miR-320a binds the 3′-UTR of the immune inhibitory receptor ligand *PDL1* and, consistently, miR-320a modulation affects PDL1 levels in MPM cells. Finally, we showed that p53 over-expression induces the upregulation of miR-320a, along with miR-200a and miR-34a, both known to target *PDL1*, and reduces PDL1 levels in MPM cells. Our data suggest that PDL1 expression might be due to a defective p53-regulated miRNA response, which could contribute to MPM immune evasion or tumorigenesis through tumor-intrinsic roles.

## Introduction

Malignant mesothelioma (MM) is a highly aggressive tumor associated with exposure to asbestos. Over 30,000 new cases of MM and over 25,000 associated deaths were estimated to occur worldwide in 2018^1^, contributing importantly to the global cancer burden. Incidence and mortality rates show high regional variability reaching epidemic proportions in some locations^2^. Indeed, higher rates of MM are predominant in areas characterized by an occupational exposure to asbestos, which is documented in ~70% of cases; whereas non-occupational exposure (owed to cohabitation with occupationally exposed subjects) or environmental exposure have been documented in 4.9% and 4.4% of cases, respectively^3^. MM is characterized by a long latency so, despite asbestos use has been banned in most countries, some studies predict an increase in incidence in some areas^4,5^.

Various studies identified genetic factors that confer susceptibility to MM, highlighting the need for genetic testing in some patients and their relatives, which could result in earlier diagnosis or provide the opportunity for possible selective treatments. It is estimated that at least 12% of MMs develop in carriers of genetic mutations: beyond *BAP1*, which is found inactivated by both germline and somatic mutations in MM, other tumor suppressor genes, such as *TP53* and *BRCA2*, or genes that regulate DNA repair, bear germline mutations in MMs^5,6^.

Most MMs affect the pleura. Malignant pleural mesotheliomas (MPMs) are classified into three main histological subtypes: epithelioid, sarcomatoid, and biphasic^7^. Epithelioid MPMs have a better prognosis compared with sarcomatoid tumors, whereas for the biphasic histotype the prognosis worsens with the increasing amount of the sarcomatoid component^5,8^. All MPMs, however, determine a poor outcome. Indeed, despite multimodal treatment, the median survival for patients with unresectable disease is limited to 9–12 months and to 17–25 months for patients with resectable MPM^5,8,9^, whereas chemotherapy including cisplatin and an antifolate (pemetrexed or raltitrexed) increases survival to approximately 12 months^9^. Various factors contribute to MPM poor prognosis. No valid biomarkers in the clinical practice allow an early detection and so the disease is usually diagnosed at a late stage; no current modality is curative, not even trimodal treatments including surgery, chemio and radiotherapy^10^; despite encouraging preclinical studies, MPM in the clinical setting is refractory to most targeted therapies attempted so far^11^.

MicroRNAs (miRNAs) have a key role in regulating gene expression, underlying most biological processes^12^. Many studies focused on identifying miRNA function in cancer, evaluating the possible use of specific miRNAs, or miRNA signatures, as diagnostic, prognostic, or predictive factors in tumors, including MPM (extensively reviewed recently^13–17^). Here, we performed a microarray screening to identify miRNAs that are differentially expressed between normal mesothelium and MPM of the three main histotypes with the aim of exploring the contribution of specific miRNAs to mesothelial tumorigenesis, possibly identifying new disease biomarkers and potential therapeutic targets.

## Materials and methods

### Patient specimens

Formalin-fixed paraffin-embedded (FFPE) MPM specimens were retrieved from the Azienda Ospedaliera Universitaria Senese (AOUS) archives and collected for retrospective analysis following AOUS ethic committee approval. All haematoxylin/eosin and immunohistochemistry slides were reviewed by two pathologists; cases of uncertain diagnostic definition and diagnostic biopsies were excluded, whereas specimens obtained from extensive tumor resection, having histological features representative of the neoplastic mass warranting certainty of diagnosis were included in the study. A total of 32 specimens were selected, of which 14 were chosen for miRNA microarray analysis (screening set) and 18 for subsequent analysis through real-time qRT-PCR (extended series); patients features are reported in Supplementary Table 1.

Normal pleural FFPE controls consisted of small flaps of mediastinal pleura excised for histological analysis during aortocoronary-bypass surgery from individuals of same age range. Specimens showing high amounts of inflammatory infiltrate, mesothelial reactive changes or insufficient preservation of mesothelial cells at microscopic observation were ruled out; only tissues that were non-pathologic and rich in mesothelial component upon histologic examination were included.

### RNA extraction for miRNA microarray analysis

Multiple 20 μm consecutive FFPE sections were cut and put on glass slides; tumor tissues were macrodissected under a stereomicroscope to remove non-neoplastic tissue and areas of necrosis and/or inflammation, whereas sections of normal pleura were deprived of subpleural fat and enriched with the thin layer of mesothelial cells. Total RNA was extracted from macrodissected sections using RecoverAll Total Nucleic Acid Isolation Kit for FFPE tissues (Ambion).

### MiRNA microarray analysis

MiRNA expression in FFPE MPM and normal mesothelial specimens was analyzed through the miRCURY™LNA™Array microRNA Profiling Service (Exiqon). Array data have been deposited in the ArrayExpress database at EMBL-EBI (www.ebi.ac.uk/arrayexpress) under accession number E-MTAB-8790. Briefly, 450 ng of RNA, following quality control, were labeled with the Hy3™-fluorescent label through the miRCURY™LNA™Array power labeling kit. A Hy5™-labeled common reference, against which each individual sample could be tested to assay biological variations among the specimens, was constituted by pooling equal amounts of RNA from each tumor and control. Hy3™-labeled and the Hy5™-labeled samples were mixed pair-wise and hybridized to the miRCURY™LNA™array version 11.0 (Exiqon). The hybridization, scanning, and normalization were performed as indicated www.ebi.ac.uk/arrayexpress. Log2-transformed median Hy3/Hy5 ratios (LMR) were obtained using median fluorescent signals on capture probe replicates; average LMRs were calculated across groups. Differentially expressed miRNAs were identified by calculating the difference in average LMR (ΔLMR) between sample groups versus normal group and subjected to ANOVA, using *p*-value < 0.001.

### Cell cultures

MSTO-211H (ATCC^®^CRL-2081™), NCI-H28 (ATCC^®^CRL-5820™) and NCI-H2052 (ATCC^®^CRL-5915™) mesothelioma and HEK-293 cell lines (ATCC^®^CRL-1573™), were recently purchased from ATCC; IST-MES2 from the ISTGE Cell Repository. All cell lines were maintained in RPMI1640 (SigmaAldrich) except IST-MES2 and HEK-293, which were grown in DMEM (SigmaAldrich), with standard supplements and conditions and periodically tested with the PlasmoTest™—Mycoplasma Detection Kit (Invivogen, for the presence of mycoplasma which was eradicated with Plasmocin™—mycoplasma elimination reagent (Invivogen, Cat# ant-mpt) when necessary.

### Real-time quantitative reverse transcription (qRT)-PCR

Total RNA was extracted using the RNeasy MiniKit (Qiagen). For *PDL1* and *TP53* mRNA expression analysis, 500 ng total RNA were retrotranscribed using the iScript cDNA Synthesis kit (Bio-Rad). QRT-PCR was performed using the PowerSybrGreenMix (AppliedBiosystems). Primer sequences are listed in Supplementary Table 2. Gene expression was normalized to β*-actin* gene expression. To quantify miRNA levels, 25 ng total RNA were retrotranscribed using the miRCURY™LNA™Universal cDNA Synthesis kit. QRT-PCR was performed using the miRCURY™LNA™SybrGreenPCR master mix and miRNA-specific primers for miR-320a, miR-34a, miR-200a, and for the RNA, U6 small nuclear 1 (*RNU6-1*), which was selected and used as internal control for miRNA expression normalization (all primers and reagents were from Exiqon). Gene or miRNA expression was calculated using the 2^−ΔΔct^ method relatively to controls^18^.

### Plasmids, site-directed mutagenesis, and transfection reagents

The *PDL1* 3′-UTR region (NCBI-Reference-Sequence NM_014143.3, nt 2751–2772) containing the putative miR-320a-binding site (or its two-base mutant, nt 2767 G → C and 2768 C → G) were directionally cloned into pmirGLO Dual-Luciferase miRNA-Target Expression Vector *Pme*I/*Xba*I sites (Promega). The mutant 3′UTR (*PDL1* Mut=G2767C; C2768G) was generated through the QuikChange^®^Site-Directed Mutagenesis Kit (AgilentTechnologies). Mimic-miR320a and mimic-scramble (mimic-miRscr) (ThermoScientific) were used for transient miR-320a-overexpression. For miR-320a silencing, a previously described sponge320a p*Silencer*5.1 expressing-vector was used^19^. For miR-320a stable expression, the miRNA precursor was cloned into pmRi-ZsGreen1miRNA *BamHI/HindIII* sites (Clontech), upon annealing of the primers listed in Supplementary Table 2.

Full-length wt p53-expressing pCEFL-HA was previously described^20^. Transfections were performed using TransIT^®^-2020 for mesothelioma (Mirus Bio LLC) and Lipofectamine™2000 (ThermoScientific) for HEK-293 cells.

### Generation of mesothelioma cell lines stably over-expressing or silenced for mir320a

To generate miR-320a-silenced stable clones, MSTO-211H were transfected with p*Silencer-*sponge320a or p*Silencer-scramble* (p*Silencer-*scr) followed by selection with puromycin (SigmaAldrich) at a previously optimized concentration of 5 µg/ml. Individual stable clones were analyzed by qRT-PCR for miR-320a-silenced expression. Similarly, MSTO-211H stable clones over-expressing miR-320a were generated upon transfection with pmRi-ZsGreen1/miR-320a and pmRi-ZsGreen1/miR-NC control, co-transfected with the linear puromycin-expressing cassette (Clontech).

### Luciferase reporter assay

HEK-293 were cotransfected with mimic-miR320a or mimic-miRscr (50 nM) together with pmirGLO-*PDL1* 3′UTR (2 μg) containing either the wt or the mut miR-320a-binding site. After 48 h, luciferase activity was analyzed through the Dual-Luciferase Reporter Assay System (Promega). Firefly luciferase activity was measured using the VictorX2 Multilabel Plate Reader (PerkinElmer) and values were normalized against those of the Renilla luciferase used as internal control.

### Protein extraction, western blotting, and antibodies

Protein extraction and western blot analyses were carried out according to standard procedures. Total protein samples (50 μg) were separated through 10% SDS–PAGE and transferred to nitrocellulose membranes. Anti-PDL1 (ABF133; MerkMillipore), anti-p53, and anti-GAPDH (DO-1 Sc-126 and Sc-25778; SantaCruzBiotechnology) primary antibodies were used. Signals were detected through ECL (Millipore).

### Growth curve analysis

For growth curve analysis, 5 × 10^3^ cells were plated in 60 mm tissue culture dishes. Cell counts were performed every 48 h for 6 days using a Burker chamber. Time zero was defined as 24 h after seeding.

### MTS assay

Cells were seeded in 96-well plates at a density of 1000 cells/well in 100 μl of complete medium. Cell viability was measured at 24, 48, and 72 h after seeding through the MTS colorimetric assay, Cell Titer 96^®^ AQueous Non-Radioactive Cell Proliferation Assay kit (Promega). Briefly, cells were incubated at 37 °C for 2 h following addition of the MTS solution (20 μl/well) to the cell medium; then the absorbance was detected at 490 nm with a 96-well plate reader (Biorad).

### Immunofluorescence microscopy

Cells were seeded on glass coverslips and fixed with 4% paraformaldehyde. After 30 min of blocking in 1% BSA at RT, samples were incubated with PDL1 antibody for 1 h at 37 °C. PDL1 was visualized using an AlexaFluor555-conjugated goat anti-rabbit antibody through a fluorescence microscope (NIKON). Nuclei were visualized through hoechst staining.

### Scratch-wound assay

Cells were kept confluent for 24 h then the cell monolayer was scraped with a 200μl-micropipette tip, and gently washed in PBS1×. Images of the wound area in three random fields were captured by microscope (×100 magnification) at the indicated time points. Wound widths were measured and migration rate was calculated through the formula: migration rate = (D0−D1)/D0, with D0 and D1 representing the wound width at 0 and 24 h, respectively.

### Target site inhibition assays

To silence miR-320a, miR-34a, and miR-200a activity on *PDL1* 3′UTR, three specific target site blockers (TSBs) in a ~500 bp region were designed by Exiqon (EX48000100; Supplementary Table 2). TSBs and the Negative Control Target Protector (TSBK) were transfected alone or in combination into MSTO-211H and miR-320a over-expressing cl9 cells at equimolar ratios. Forty-eight hours post-transfection, total proteins and RNA were extracted to measure PDL1 levels.

### Statistics

Statistical analyses were performed using the GraphPad Software 5.01 and are specifically detailed in each figure legend.

## Results

### Microarray identification of differentially expressed miRNAs

To identify differentially expressed miRNAs between normal mesothelium and MPMs of different histotype, we profiled miRNA expression in archival specimens from a cohort of patients and controls, through the miRCURY Exiqon LNAarray v11 (including a total of 1253 miRNAs, of which 829 miRNAs annotated to miRBase13.0 and 423 miRPlus, which were not-yet-annotated at the time of screening). RNAs were extracted upon macrodissection from a test series of 14 FFPE MPM samples (6 epithelioid, 4 sarcomatoid, and 4 biphasic tumors), and 6 normal mediastinal pleural samples of individuals with a non-pulmonary disease. Principal component analysis (PCA) of the array results showed that MPMs express a clearly distinct miRNA profile compared with the normal pleural mesothelium. Across the tumor set, the epithelioid and sarcomatoid histotypes showed a different profile although without a complete separation; conversely, the biphasic histotypes showed a profile consistent with their histologically ‘mixed’ nature (Supplementary Fig. 1A). A total of 329 miRNAs were detected in all the specimens analyzed including tumors and normal tissues. MiRNA expression in all tumors versus normal identified 22 of 1253 miRNA with *p*-values < 0.0001 (Supplementary Fig. 1B).

**Fig. 1:**
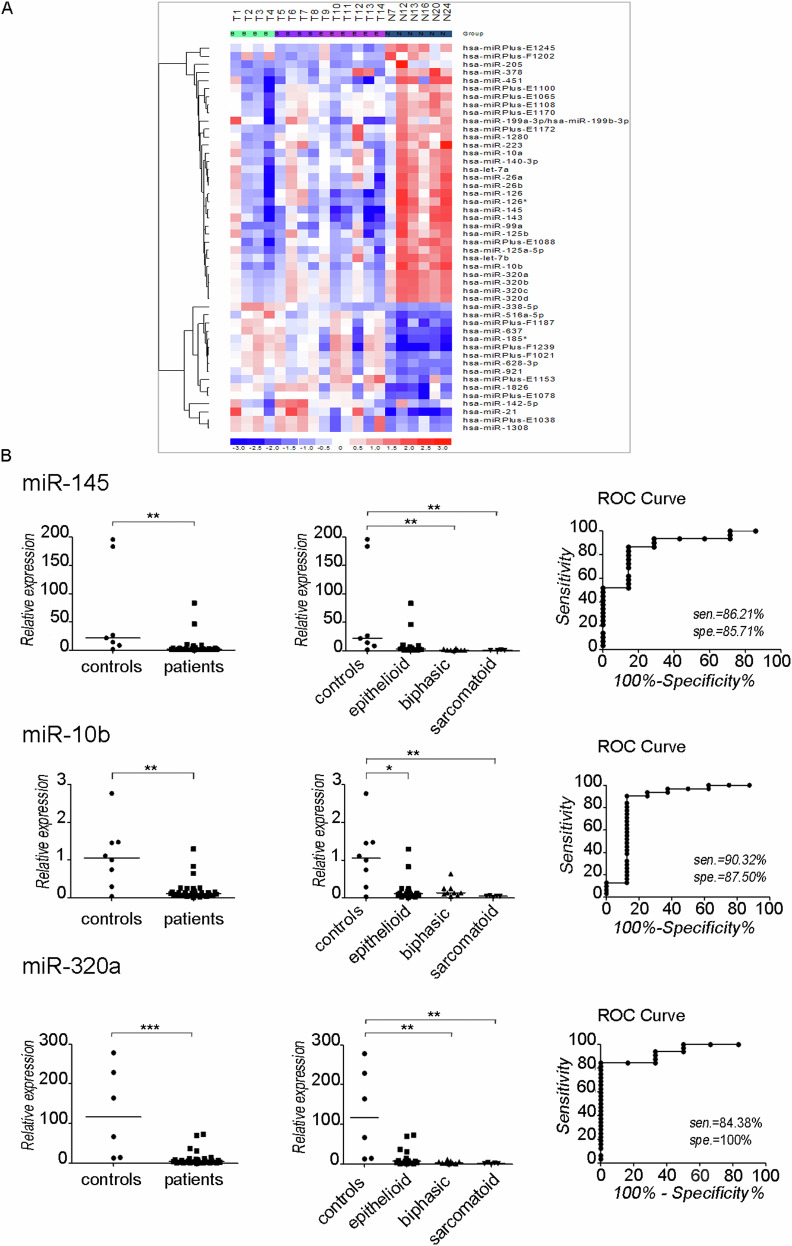
miRNA expression profiling in MPM. **a** Heat map and hierarchical clustering. The heat map diagram shows the result of the two-way hierarchical clustering of miRNAs and samples. Each row represents a miRNA and each column represents a sample. The miRNA clustering tree is shown on the left whereas the sample clustering tree is shown at the top. The clustering is performed on log2(Hy3/Hy5) ratios which passed the filtering criteria on variation across samples^69^: ∆LMR>1.5 was set for comparison to the group “normal”. The color scale shown at the bottom illustrates the relative expression level of a miRNA across all samples: the ΔLMR has been converted to fold change; a fold change > 1 (red) indicates up-regulation in tumor samples and a fold change < 1 (blue) indicates down-regulation in tumor samples compared to normal samples. Mir-plus sequences and their correspondence to recently annotated miRNAs are reported in Supplementary Table 3. **b** MiR-145, miR-10b, and miR-320a expression in MPM. The graphs show that the array results were confirmed for miR-145, miR-10b, and miR-320a also by real-time qRT-PCR and by extending the case series with other tumors. For each miRNA, the relative expression in all tumor and normal samples was calculated with respect to the same sample, used as a calibrator, by the 2^−ΔΔct^ method^18^. The dot plots on the left show the miR-320a, miR-10b, and miR-145 expression both individually for each normal and tumor sample (symbols) and as median values (lines). Statistically significant differences were evaluated through the Mann–Whitney test. The central graphs show differential miR-320a, miR-10b, and miR-145 expression levels in tumors of the three main MPM histological types. Data were subjected to Kruskal–Wallis test with Dunn post hoc test. The ROC curves on the right were constructed to examine the diagnostic accuracy of miR-145, miR-10b, and miR-320a mRNA expression. A specific pair of sensitivity and specificity was identified as the optimal cutoff threshold. The ROC curves show that the area under the curve (AUC) of miR-145 is 0.8818 (95% CI: 0.7455–1.018), of miR-10b is 0.8629 (95% CI: 0.6643–1.061) and of miR-320a is 0.9375 (95% CI: 0.8574–1.018); the diagnostic sensitivity and specificity are reliable for all the three miRNAs, being over 80%. Statistically significant differences are indicated as follows: *p < 0.05, significant; **p < 0.01, very significant; and ***p < 0.001, extremely significant.

To evaluate miRNA expression differences in epithelioid, sarcomatoid, and biphasic versus the normal mesothelium group, miRNAs were sorted based on a difference in average log median ratio (∆LMR) > 1.5. This analysis identified 48 differentially expressed miRNAs (16 upregulated and 32 downregulated in MPM versus normal mesothelial tissue), including both annotated miRNAs and miRPlus. These 48 miRNAs are shown in the two-way hierarchical clustering in Fig. 1a, miRPlus sequences are reported in Supplementary Table 3. A subset of these differentially expressed miRNAs enclosed various miRNAs previously identified in MPM, including miR-126, miR-143, miR-145^21–23^, and miR-21^23^.

We then investigated by qRT-PCR the expression of a subset of differentially expressed miRNAs, to confirm the reliability of the array results. In particular, we tested the expression of miR-145, miR-10b, and miR-320a, (which were retrieved in both ranking lists, Fig. 1a, Supplementary Fig. 1B). Indeed, we found miR-145, miR-10b, and miR-320a as differentially expressed in tumors versus controls (*p* value of 0.0020, 0.0233, and 0.0026, respectively, as determined through Mann–Whitney test). Then, we further extended the analysis of miR-145, miR-10b, and miR-320a expression by assessing through qRT-PCR a wider series of cases and controls. Downregulation of all three miRNAs in MPMs compared with controls, and their differential expression across histotypes, were confirmed in the extended case series (Fig. 1b). Also, ROC curve analysis showed that miR-145, miR-10b, and miR-320a expression levels can discriminate between tumor and control samples with high specificity and sensitivity (Fig. 1b), therefore qualifying as potential diagnostic biomarkers according to the International Mesothelioma Interest Group criteria^24^.

### MiR-320a affects MPM cell proliferation and migration

**Fig. 2:**
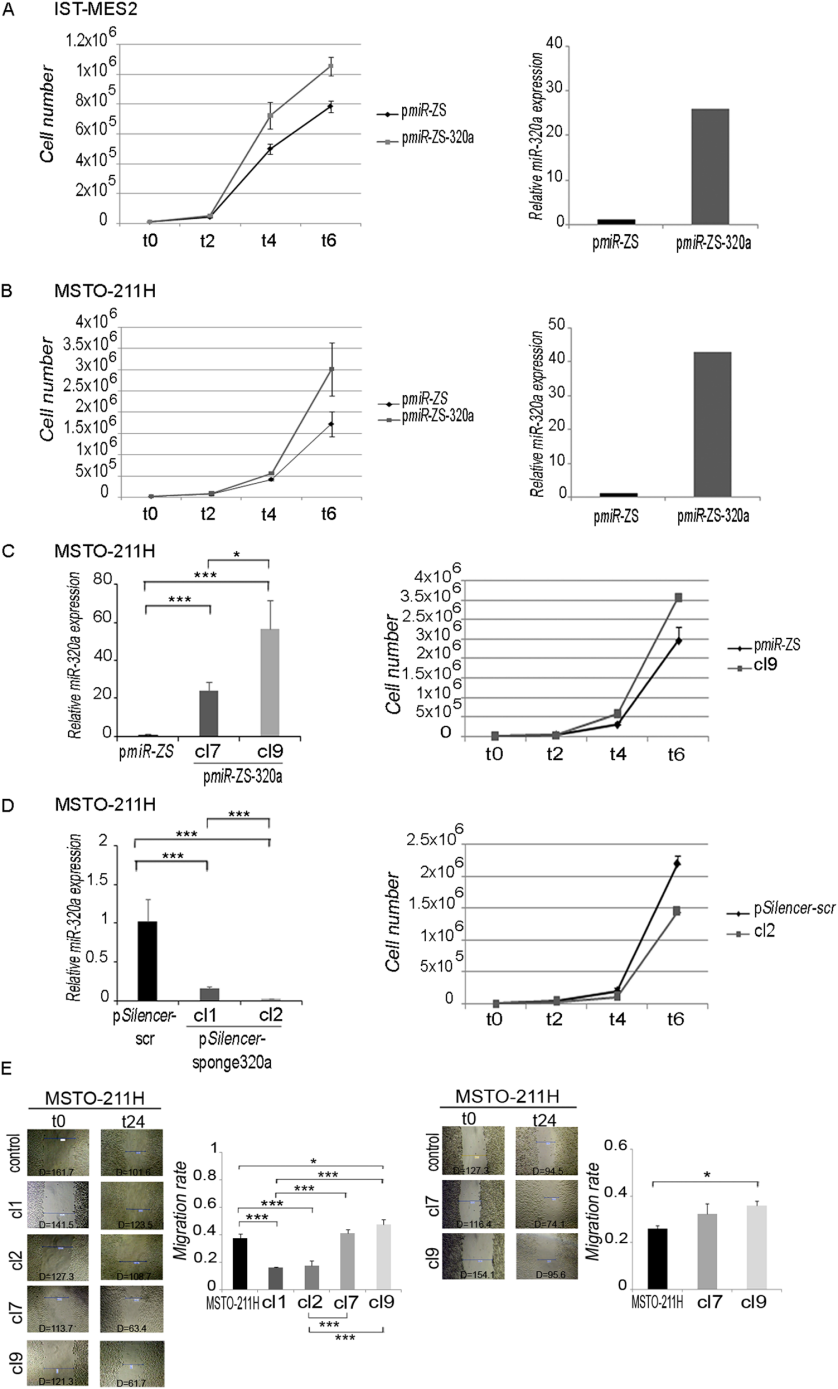
miR-320a affects MPM cell proliferation and migration. Growth curves of IST-MES2 **a** and MSTO-211H **b** cell populations stably transfected with a miR-320a expressing vector (pmiRZS-320a) or the empty vector as control (pmiR-ZS). Cells were counted every two days over a 6-day time span. The cell growth graphs report the mean number of cells with SD of five independent experiments. The curves resulted significantly different from their respective controls, as evaluated by two-way ANOVA: *p* < 0.0001 for IST-MES2 and *p* = 0.0012 for MSTO-211H, respectively. MiR-320a expression in each cell population was analyzed by real-time qRT-PCR. The miRNA levels were normalized to those of the RNA encoded by the U6 small nuclear 1 (*RNU6-1*) gene and calculated by the 2^−ΔΔct^ method relatively to control cells. Single MSTO-211H cell clones stably expressing miR-320a **c**, or silenced for its expression with a sponge construct **d**, were selected and tested for miR-320a expression by real-time qRT-PCR as described in the text. Histograms reporting miR-320a expression (mean ± SD) in three independent analyses are shown on the left. Statistically significant differences between miR-320a expression in stable clones were evaluated by one-way ANOVA with Tukey post-test and are indicated as follows: *p < 0.05, significant, and ***p < 0.001, extremely significant. Cell growth curves of MSTO-211H clones expressing higher (cl9) or lower (cl2) miR-320a levels, respectively, are shown on the right and report data from three independent analyses. The curves resulted significantly different from their respective controls, as evaluated by two-way ANOVA, *p* = 0.0022 for cl2 and *p* = 0.0046 for cl9, respectively. **e** Effect of miR-320a modulation on MSTO-211H cell migration ability. Cell migration capacity of stable MSTO-211H clones, silenced (cl1 and cl2) or over-expressing miR-320a (cl7 and cl9), was evaluated through a scratch-wound assay and compared with that of MSTO-211H parental cells (control), left panel. Representative images of the wound area captured by microscope (×100 magnification) at the indicated time points (0 and 24 h) are shown, whereas the graph reports the means ± SD of the migration rate from three independent experiments. The right panel reports representative images of cl7 and cl9 clones compared with MSTO-211H parental cells tested in low serum conditions. The graph reports the means ± SD of the migration rate from three independent experiments. Statistically significant differences between stable clones and parental cells were evaluated by one-way ANOVA with Tukey post-test (*significant *p* < 0.05, ***extremely significant *p* < 0.001).

We then specifically focused on miR-320a because its role in MPM has not been investigated yet and we were intrigued by the fact that other miRNAs of the miR-320 family (miR-320b, 320c, and 320d) were similarly downregulated in MPMs. As these miR-320 family members are located on different chromosomes we hypotesize that their downregulation is a regulated (non-casual) event underlying mesothelial tumorigenesis. We set out to assess the effect of miR-320a in MPM cell lines, expecting that it would function as a tumor suppressor, similarly to other tumor types in which it was also found downregulated. To this purpose, we generated IST-MES2 and MSTO-211H stably expressing the miR-320a precursor (pmiR-ZS-320a). Upon transfection, selection, and verification of miR-320a-expression levels (Fig. 2a, b), we evaluated miR-320a effect on IST-MES2 and MSTO-211H cell population proliferation rate. Surprisingly, miR-320a stable over-expression increased rather than decrease the proliferation rate in both cell lines (Fig. 2a, b). We verified such effect by analysing growth of single miR-320a-stably expressing clones. In particular, we screened two miR-320a-stably expressing MSTO-211H clones (cl7 and cl9) and analyzed the growth of cl9, which showed higher miR-320a expression (Fig. 2c). Indeed, miR-320a increased cell proliferation rate slightly but significantly. Conversely, miR-320a downregulation decreased MSTO-211H proliferation, as assessed in clone cl2, stably expressing a sponge320a construct, which achieved strong miR-320a silencing (Fig. 2d). Consistently, an MTS analysis showed that miR-320a stable expression in cl7 and cl9 cells increased cell viability compared with parental cells, whereas miR-320a stable silencing in cl1 and cl2 decreased cell viability (Supplementary Fig. 2).

We then evaluated how the modulation of miR-320a expression impacted on MPM cell ability to migrate, through a scratch-wound assay. To minimize the effect of proliferation on wound closure, cells were kept confluent and migration was evaluated early, at 24 h. MiR-320a-over-expressing MSTO-211H cl7 and cl9 cells showed a higher migration ability, although statistical significance was reached only for cl9 compared with parental cells. Conversely, miR-320a-silenced cl1 and cl2 showed a decreased migration rate (Fig. 2e, left panel). Cl7 and cl9 were also assayed in 1% FBS, because were tolerant to this condition, differently from cl1 and cl2. MiR-320a over-expressing cells were confirmed to have increased migration ability compared with MSTO-211H parental cells also in low serum (Fig. 2e, right panel).

### MiR-320a directly targets PDL1

**Fig. 3:**
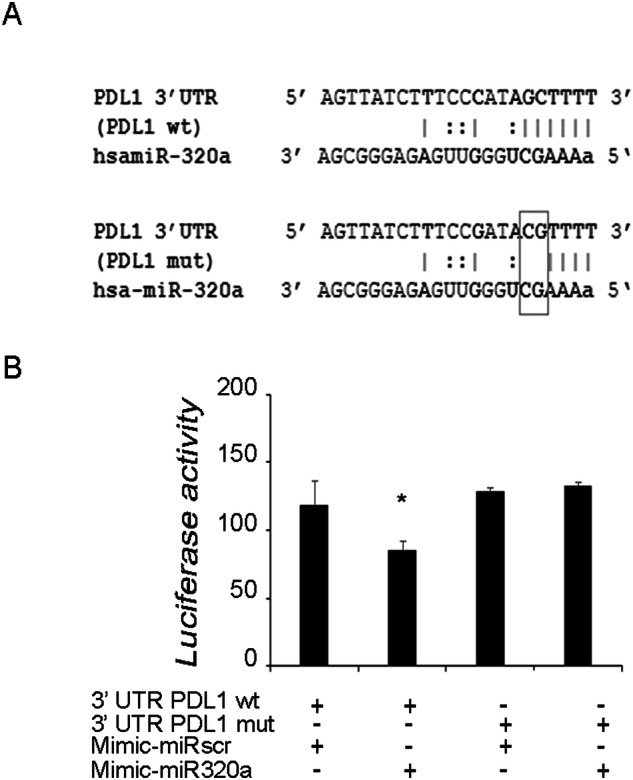
PDL1 is a direct target of miR-320a. **a** The putative miR-320a binding site within *PDL1* 3′ UTR, as predicted through the Segal lab online software, with the highest score, is indicated. The alignment of both wt or mutant miR-320a and the *PDL1* 3′ UTR (NCBI reference sequence NM_014143.3, nt 2751–2772) are shown; the mutated bases are included in a box. **b** A luciferase reporter assay was performed in HEK-293 cells 48 h after cotransfection with the wt or mutated pmirGLO-*PDL1* 3′UTR miR-320a binding site plasmids in the presence of mimic-miR-scr (scramble) or mimic-miR-320a. For each sample firefly luciferase activity values were normalized to those of Renilla luciferase activity, used as an internal control. The graph reports the means ± SD from three independent experiments. Statistically significant differences between various conditions were evaluated by one-way repeated-measures ANOVA with Tukey post-test. Luciferase activity in cells cotransfected with wt *PDL1* 3’UTR and mimic-miR-320a resulted significantly different from that of all the other conditions (*significant *p* < 0.05).

Despite miR-320a was found downregulated in MPM specimens, its modulation in MPM cell lines did not seem to support a ‘classic’ role as tumor suppressor miRNA. So, we wondered whether miR-320a could affect other mechanisms, not directly related to tumorigenic cell features like proliferation or migration. Among miR-320a putative targets, we identified *PDL1* (CD274) through the prediction tool https://genie.weizmann.ac.il/pubs/mir07/mir07_prediction.html^25^. Putative miR-320-binding sites were found within *PDL1* mRNA. To confirm miR-320a and *PDL1* interaction, we cloned, downstream the luciferase reporter, the wt (or mutated) binding site within *PDL1* 3′-UTR, which was predicted with the highest score (Fig. 3a). We then evaluated luciferase activity 48 h after HEK-293 transfection with the wt or mutated binding site, along with either miR-320a mimics or scramble control (Fig. 3b). The co-transfection of the *PDL1*wt site-containing vector with mimic-miR320a significantly reduced luciferase activity. Conversely, no repression of luciferase activity was observed when miR-320a mimics were co-transfected with the vector containing the mutated 3′UTR. The slightly higher basal luciferase activity of the mutated-site-containing vector is likely due to the impaired binding of endogenous miR-320a. In summary, these results support that *PDL1* 3′UTR is a direct target of miR-320a.

**Fig. 4:**
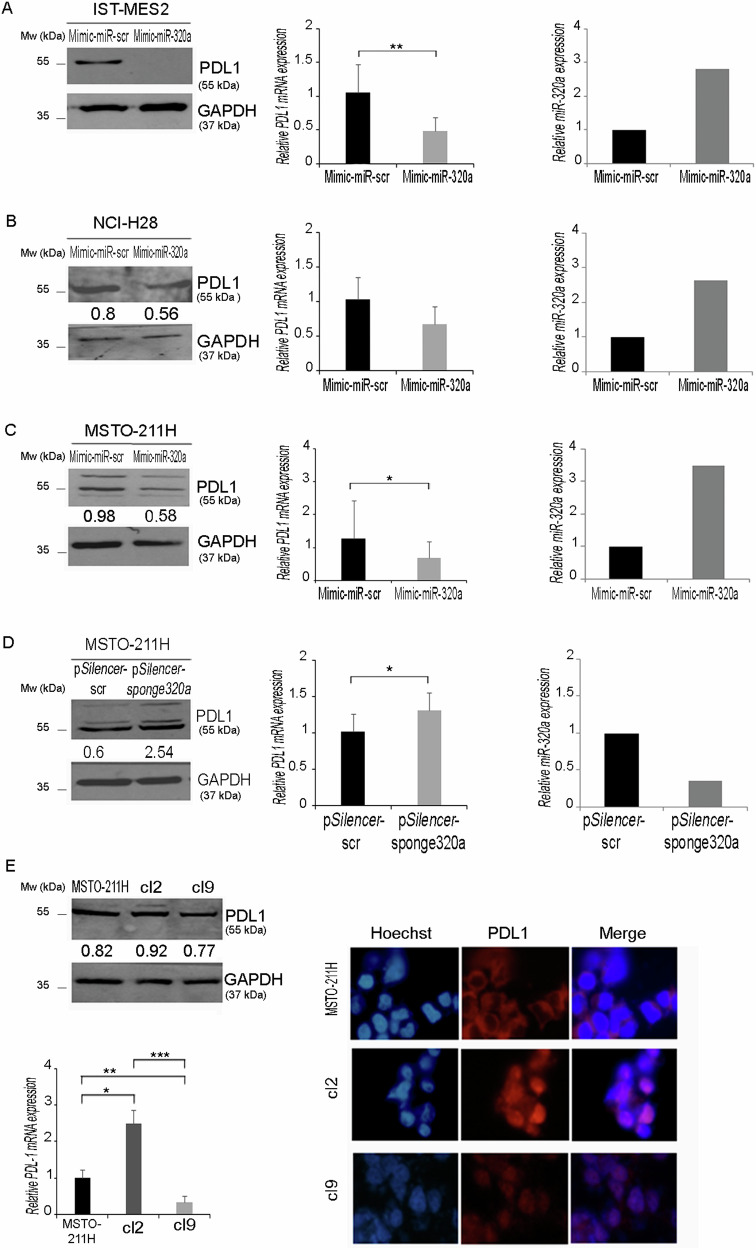
miR-320a effect on PDL1 expression in MPM cell lines. IST-MES2 **a**, NCI-H28 **b**, and MSTO-211H **c** were transfected for 48 h with mimic-miR-320a or mimic-miR-scr as a control. A representative experiment showing PDL1 protein expression analyzed by western blot is shown on the left. The intensity of the bands was quantified by densitometric analysis and PDL1 band densities were normalized against GAPDH. The *PDL1* mRNA expression was analyzed by real-time qRT-PCR and normalized with respect to β-actin expression. Histograms report *PDL1* relative expression (mean ± SD) from three independent analyses. Paired two-sided Student’s *t*-test was used to analyze the significant differences: *significant (*p* < 0.05) and **very significant (*p* < 0.01). MiR-320a relative expression for each cell line is shown in the histograms on the right, as a transfection control. **d** The effect of miRNA silencing was tested in MSTO-211H cells transiently transfected with the pSilencer-sponge320a construct or its control. MiR-320a and PDL1 levels were analyzed as described above. Paired two-sided Student’s *t*-test was used to evaluate statistically significant differences (*significant, *p* < 0.05; *n* = 3). **e** PDL1 mRNA and protein levels were also tested by real-time qRT-PCR, western blot and immunofluorescence in the single MSTO-211H clones over-expressing (cl9) or silenced (cl2) for miR-320a. Real-time qRT-PCR results represent the means and SD of three independent experiments. Statistically significant differences between stable clones and parental cells were evaluated by one-way ANOVA with Tukey post-test (*significant, *p* < 0.05; **very significant, *p* < 0.01; ***extremely significant, *p* < 0.001). A representative image of two immunofluorescence analysis is shown.

We then assessed the effect of miR-320a on PDL1 expression levels in various MPM cell lines including IST-MES2, NCI-H28, and MSTO-211H. The introduction of a miR-320a mimic led to a decrease of the PDL1 mRNA and protein levels (Fig. 4a–c). To assess whether the effect on PDL1 was indeed miR-320a-specific, we co-expressed, along with the miR-320a mimic, a TSB specific to miR-320a (TSB 320a) or a control sequence, TSB K. Owing to their higher affinity, TSBs outcompete miRNA binding, preventing mRNA-target translational attenuation. The addition of TSB 320a counteracted the suppressive effect of miR-320a on both PDL1 mRNA and protein expression (Supplementary Fig. 3).

Consistently, silencing endogenous miR-320a levels in MSTO-211H cells, by transient transfection of the pSilencer-sponge320a, led to increased PDL1 mRNA and protein levels (Fig. 4d).

We also confirmed miR-320a and PDL1 inverse relationship in the stable MSTO-211H clones, either over-expressing miR-320a (cl9), or silenced for its expression (cl2). Higher *PDL1* mRNA levels were observed in miR-320a-silenced cl2 relatively to parental cells, whereas lower expression levels were observed in the miR-320a-over-expressing cl9 cells. A milder effect was observed at the protein level by western blot, which was consistent also through immunofluorescence analysis (Fig. 4e). These results further confirmed a functional link between miR-320a and PDL1 in mesothelioma cell lines.

### MiR-320a targeting of PDL1 is likely part of a p53-mediated response, which includes also miR-34a and miR-200a

**Fig. 5:**
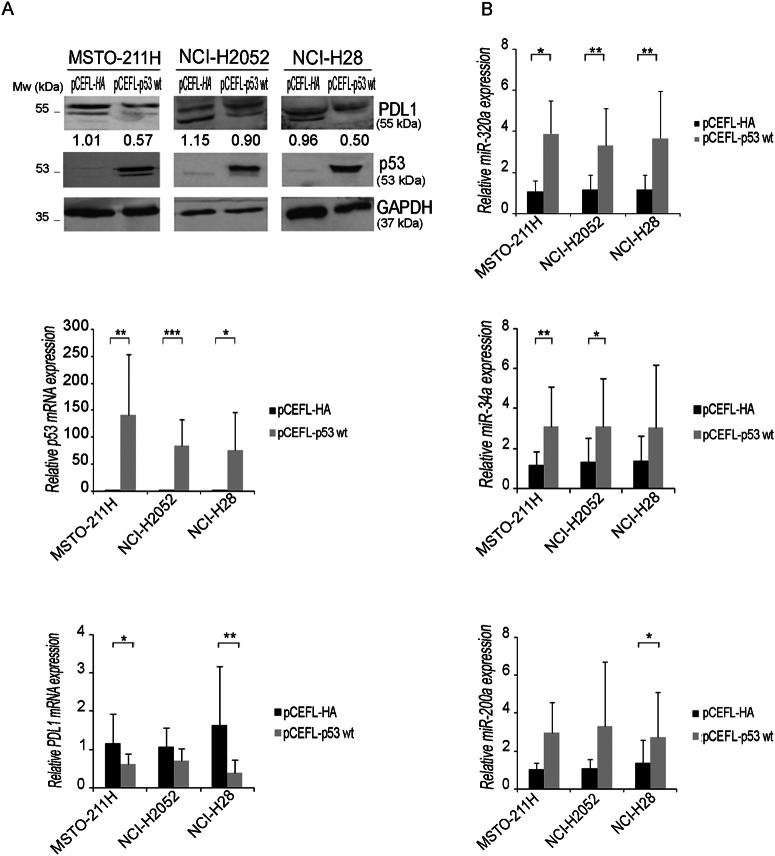
p53 effect on PDL1 and miRNA expression in MPM cell lines. **a** MSTO-211H, NCI-H2052, and NCI-H28 were transfected with a p53 wt expression vector or its pCEFL-HA-empty vector as control. Forty-eight hours upon transfection, cells were lysed and collected for protein analysis. Western blots confirmed that p53 overexpression suppressed PDL1 protein levels compared with their respective controls. A blot with an anti-p53 antibody was used to control transfection levels, whereas an anti-GAPDH antibody was used as a loading control. A representative western, out of three independent ones, is shown. *TP53* and *PDL1* mRNA relative expression was analyzed by real-time qRT-PCR in all MPM cell lines transiently transfected with p53 wt. Paired two-sided Student’s *t-*test was used to evaluate statistically significant differences (*significant, *p* < 0.05; **very significant, *p* < 0.01; ***extremely significant, *p* < 0.001; *n* = 3). Error bars indicate SD. **b** The expression of miR-320a, miR-34a, miR-200a in MSTO-211H, NCI-H2052, and NCI-H28, transfected with either p53 wt or pCEFL-HA-empty vector, was analyzed by real-time qRT-PCR. Results from at least three independent experiments are shown. Paired two-sided Student’s *t-*test was used to evaluate statistically significant differences (*significant, *p* < 0.05; **very significant, *p* < 0.01). Error bars indicate SD.

**Fig. 6:**
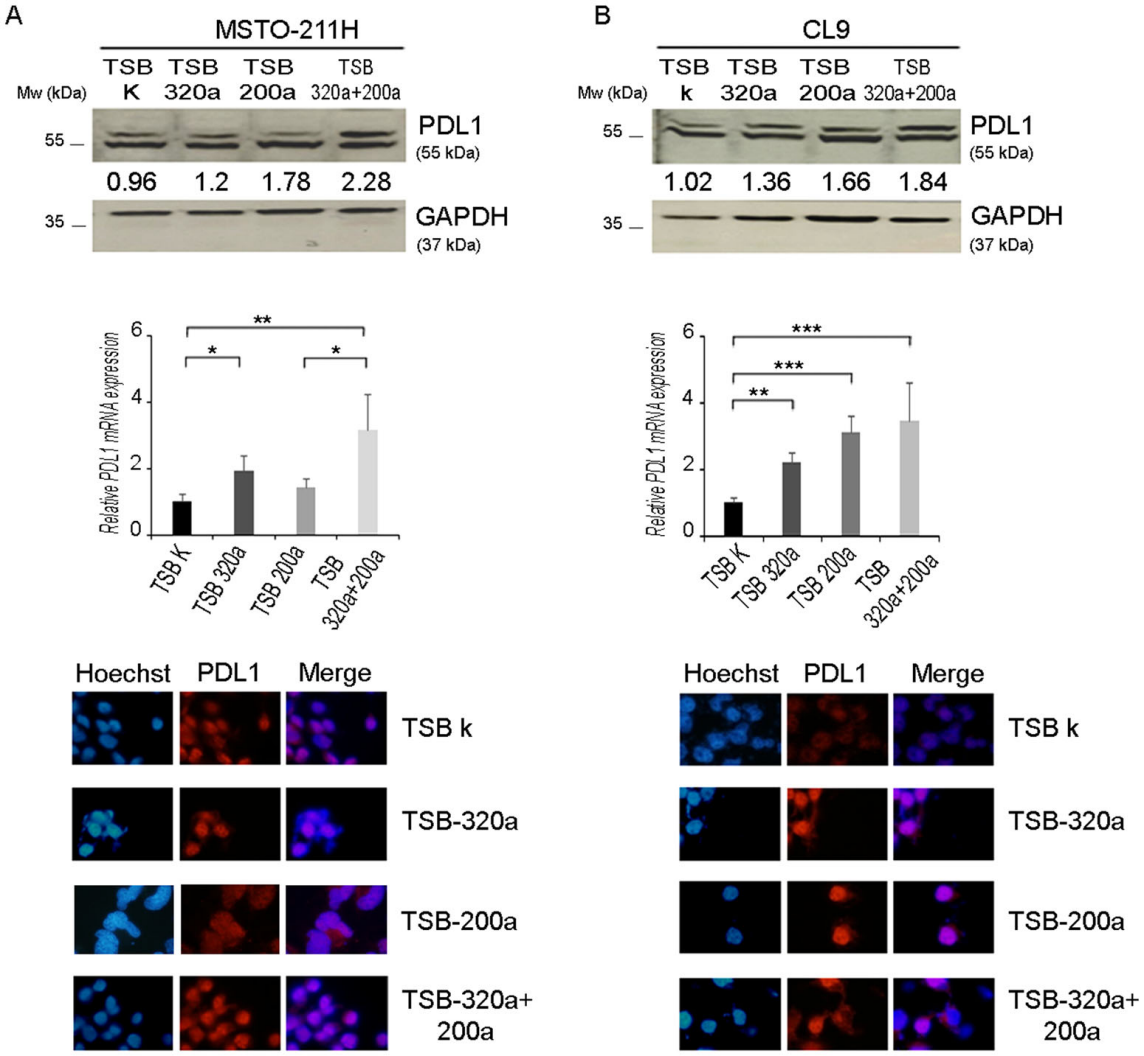
MiRNA contribution to PDL1 expression in MPM. MSTO-211H **a** and cl9 **b** were transfected with TSB-320a, TSB-200a, or their combination and with the negative control (TSB K). The expression levels of PDL1 were analyzed by western blotting with anti-PDL1 and anti-GAPDH antibodies. The intensity of the bands was quantified by densitometric analysis and PDL1 band densities were normalized against GAPDH. Real-time qRT-PCR analysis was performed to measure the *PDL1* mRNA expression and data are represented as means ± SD. Statistically significant differences were evaluated by one-way repeated-measures ANOVA with Tukey post-test (*significant *p* < 0.05; **very significant *p* < 0.01; ***extremely significant, *p* < 0.001; *n* = 3). A representative image of two independent immunofluorescence analyses is shown.

Previous studies showed that p53 regulates PDL1 levels via miR-34a and the deregulation of this axis has been proposed as a mechanism underlying tumor immune evasion in non-small cell lung cancer^26^. We previously showed that p53 can directly activate miR-320a transcription upon stress^19^. So, we asked whether p53 was involved in the miR-320a-mediated regulation of PDL1 observed in MPM cell lines. We over-expressed p53 in MPM cells and evaluated PDL1 expression. In all cell lines, p53 over-expression led to miR-320a upregulation and reduced PDL1 mRNA and protein levels (Fig. 5a, b). However, as p53 regulates miR-34a^26^ and miR-200a^27^, which also target *PDL1*^26,28^, we evaluated also their expression and found their upregulation in all MPM cells following p53 over-expression (Fig. 5b). To dissect the contribution of each specific miRNA to PDL1 regulation, we used TSBs. We transfected parental MSTO-211H and miR-320a-over-expressing cl9 with either TSBs or TSBK control and found that both TSB-320a and TSB-200a increased *PDL1* mRNA and protein levels, whereas TSB-34a did not, probably because it acts through a different binding site within *PDL1* mRNA (Supplementary Fig. 4). We then evaluated in both parental MSTO-211H and miR-320a-over-expressing cl9 the effect of the concomitant inhibition of miR-320a and miR-200a. The concomitant use of TSBs, blocking the activity of both miRNAs, achieved higher PDL1 mRNA and protein levels, the latter assessed both by western blot and immunofluorescence analysis (Fig. 6a, b). Overall, our data show that multiple miRNAs induced by p53 contribute to the complex regulation of PDL1 expression in MPM.

## Discussion

Despite much progress in defining molecular mechanisms underlying MPM tumorigenesis, providing an early and correct diagnosis still represents a challenge^5^. Given the potential of miRNAs to serve as reliable biomarkers, many recent studies focused on the identification of miRNAs differentially expressed in MPM tissues and body fluids compared with normal controls or other pathologic conditions (from benign asbestos-related pleural effusions to lung adenocarcinomas). These studies provided quite heterogeneous findings highlighting the need for a clear reporting of the study design and of any possible bias to allow confrontation in multi-institutional validations, and well-powered analyses in larger-scale datasets, often not available for this low-frequency disease^16^. Here, we set out to explore miRNA expression with the aim to uncover novel players underlying MPM tumorigenesis, tackling some of the issues raised as follows. First, we carefully selected archival FFPE MPM specimens including only samples derived from extensive tumor resection, with histological features representative of each specific histotype ruling out biopsies or cases of uncertain diagnostic definition. As the thin layer of normal mesothelial cells flakes away owing to manipulation during surgical procedures, the normal specimens were specifically handled to preserve the mesothelial cells and processed identically to the MPM specimens for a proper comparison. Both MPM and normal samples were also macrodissected to enrich for tumor content (>80%) and mesothelial cells, respectively. Fourteen MPM and six controls were selected for the discovery phase through a microarray-based miRNA expression screening. We chose an array, including 253 miRNAs, based on LNA oligonucleotides, which have high affinity for their complementary strand, resulting in high sensitivity and specificity for miRNA detection. Our analysis identified 16 upregulated and 32 downregulated miRNAs in MPM versus normal mesothelial tissue, including miRNAs previously identified in other studies, such as miR-126, miR-143, miR-145 among the downregulated ones, and miR-21, which we also found upregulated in MPMs, consistent with previous data^16,29^. To validate our data through another method, we analyzed by qRT-PCR a subset of miRNAs retrieved in both the fold-change-based and *p*-value-based rankings including miR-145, miR-10b, and miR-320a, expanding the analysis to a wider series. All three miRNAs were indeed downregulated in tumors and their expression levels discriminated between tumor and control samples with high specificity and sensitivity qualifying as potential MPM diagnostic biomarkers. In particular, these results confirmed also in our cohort that miR-145 expression levels can be used to differentiate benign versus malignant mesothelial tissue^16,21,22^.

We then focused specifically on miRNA-320a, which was recently shown to act as a tumor suppressor in different cancer types in which it was found downregulated, such as colorectal^30,31^, breast^32,33^, bladder^34^, lung^35–37^, prostate^38^ and gastric cancer^39,40^, multiple myeloma^41^ and gliomas^42–44^.

Here, we found that miR-320a was significantly repressed in MPM specimens, in particular in the most aggressive biphasic and sarcomatoid histotypes. Our microarray results revealed, among the top-ranking alterations, the simultaneous downregulation of multiple miR-320 family members, located at different chromosomes (miR-320a on Chr8, miR-320b on Chr1, miR-320c on Chr18, and miR-320d on Chr13, respectively), suggesting that decreased expression of miR-320 family is not a casual event in MPM tumorigenesis.

So, we set out to analyze the biological effect of miR-320a in MPM cell lines. However, rather than acting as a tumor suppressor, as described in other cancers, we found that miR-320a stable over-expression in IST-MES2 and MSTO-211H promoted cell proliferation. Subsequent analysis of MSTO-211H individual clones showed that miR-320a stable over-expression increased cell proliferation, viability, and migration, whereas miR-320a stable silencing decreased them. These findings are consistent with a study in which miR-320a/c/d ectopic expression increased hepatocarcinoma cell migration and invasion^45^, and a study in which miR-320a promoted proliferation, migration, invasion, and reduced pancreatic cancer cell sensitivity to chemotherpeutics^46^, suggesting that miR-320 pro-tumoral or anti-tumoral functions are context-dependent. However, miR-320a cell-autonomous roles will have to be further investigated in other MPM cell lines and models to assess whether the effects observed in IST-MES2 and MSTO-211H can be generalized or, rather, they are context-specific.

At present, only another study analyzed miR-320 expression in MPM^47^, however the authors analyzed miRNAs from frozen biopsies and confronted them with benign-asbestos-related pleural effusions rather than normal mesothelium. Further analyses of miR-320-family expression in other wide and uniform cohorts including normal tissue, MPMs, and benign pleural pathologies are necessary to establish the possible clinical utility of these miRNAs as biomarkers for MPM and other pleural conditions.

To investigate possible mechanisms whereby miR-320a status might affect mesothelioma tumorigenesis, we searched for predicted targets and identified PDL1, which endows cancer cells the ability to evade the host-antitumoral immune response. Interestingly, high expression of PDL1 in MPM predicts poorer survival and is associated to the non-epithelioid histotypes^48^, which are those with the lower expression of miR-320a in our study. Moreover, beyond immune evasion, it is emerging that PDL1 tumor-intrinsic signaling can affect cancer initiation, development, and treatment^49^. We demonstrated that miR-320a indeed regulates *PDL1* expression by targeting its 3′UTR. Consistently, we showed that miR-320a modulation in MPM cell lines of different histotypes regulates PDL1 expression. PD-L1 was found highly expressed in mesothelioma and within the tumor stroma^50^ and later associated particularly with sarcomatoid histotype and poor survival^51,52^. Current efforts are aimed at assessing whether immune checkpoint inhibitors, including agents targeting PDL1 and its receptor, can be used against MPM at least for selected patients. Therefore, studying PDL1 expression in MPM and its microenvironment, and PDL1 correlation with response to targeted immunotherapy, is a matter of intense investigation.

Recently, various miRNAs were found to regulate PDL1 in cancer, including miR-15a-miR-16^53^, miR-34^26^, miR-142-5p^54^, miR-152^55^, and miR-200a^56^. Reid and colleagues showed that, in MPM, expression of miR-15a and miR-16 tumor suppressors inversely correlated to PDL1 expression and, similarly, low levels of miR-200 family members were found in PDL1-positive tumors; however they did not find differential expression of the three members of the miR-34 family in PDL1-positive and PD-L1-negative MPMs^53,57^. Interestingly, both miR-34 and miR-200 families are controlled by p53^26,27^ and we previously found that p53 induces miR-320a in response to stress^19^, similar to the ΔNp63α family member^58^. So, we assessed whether also in MPM p53 could regulate miR-320a and indeed found that p53-ectopic expression in MPM cells increased the levels of miR-320a, and also of miR-34 and miR-200a confirming the previous observations. Concomitantly with miRNA upregulation, p53 reduced PDL1 mRNA and protein levels and, blocking miRNA suppressive function with target site blockers suggested that these p53-regulated miRNAs cooperate in regulating PDL1 expression.

P53, likely the most frequently inactivated protein in cancer^59,60^ is reported as infrequently mutated in MPMs, however its function is inactivated by common defects in its pathway, such as the *CDKN2A* deletion occurring in most MPMs^5^. More recently p53 mutations have been associated with poorer overall survival^61^. Also, we previously showed that p53-reactivating agents could be used effectively against MPM^20^. P53 has both cell autonomous and non-cell autonomous roles in the homeostatic regulation of immune responses, both established and emergent roles^62,63^. Among the latter, although a study reported increased PDL1 levels upon p53 induction in MCF7 breast cancer cells^64^, *TP53* mutations increase PDL1 levels in lung adenocarcinomas^65,66^. Similarly, also in melanoma *TP53*-mutated tumors were associated with higher PDL1 positivity, although the underlying mechanism was not transcriptional^67^.

Overall, our data, beyond identifying a new signature of miRNA differentially expressed between normal mesothelium and MPMs, reveal yet another mechanism whereby p53 can affect PDL1 expression, adding miR-320a to the list of miRNAs targeting this immune checkpoint, which are receiving increased attention^68^. It remains to be established whether the miR-320a downregulation observed in our MPM cohorts could be a consequence of p53 pathway inactivation (being a passenger rather than a driver alteration) and whether p53 mysfunction might favor tumor escape from immune surveillance. According to our data, miR-320a, while potentially serving as a useful diagnostic/prognostic biomarker for MPM, would not be a feasible therapeutic target; rather, the previously devised p53 restoring strategies could have further advantages for as concerns the anticancer immune response. Also, it will be interesting to further investigate how the high PDL1 levels often found in the most aggressive MPM subtype affect tumor-intrinsic mechanisms of tumorigenesis beyond immune evasion.

## Supplementary information

Supplementary Figure 1

Supplementary Figure 2

Supplementary Figure 3

Supplementary figure 4

Supplementary Tables

Supplementary Figure legends
