# Correction: P53-regulated miR-320a targets PDL1 and is downregulated in malignant mesothelioma

**DOI:** 10.1038/s41419-020-03016-5

**Published:** 2020-10-16

**Authors:** Caterina Costa, Paola Indovina, Eliseo Mattioli, Iris Maria Forte, Carmelina Antonella Iannuzzi, Luca Luzzi, Cristiana Bellan, Simona De Summa, Enrico Bucci, Domenico Di Marzo, Marisa De Feo, Luciano Mutti, Francesca Pentimalli, Antonio Giordano

**Affiliations:** 1 Cell Biology and Biotherapy Unit, Istituto Nazionale Tumori-IRCCS-Fondazione G. Pascale, I-80131 Napoli, Italy; 2 Sbarro Institute for Cancer Research and Molecular Medicine, Center for Biotechnology, College of Science and Technology, Temple University, Philadelphia, PA 19122 USA; 3 Institute for High Performance Computing and Networking, National Research Council of Italy (ICAR-CNR), Naples, Italy; 4 Histopathological Unit, IRCCS-Istituto Tumori “Giovanni Paolo II”, Viale Orazio Flacco 65, 70124 Bari, Italy; 5 Thoracic Surgery Unit, Department of Medicine, Surgery and Neuro Sciences, Diagnostic Imaging, University of Siena, Azienda Ospedaliera Universitaria Senese, Siena, Italy; 6 Department of Medical Biotechnologies, University of Siena, Siena, 53100 Italy; 7 Molecular Diagnostics and Pharmacogenetics Unit-IRCCS-Istituto Tumori “Giovanni Paolo II”, Viale Orazio Flacco 65, 70124 Bari, Italy; 8 Department of Cardiothoracic Sciences, Università degli Studi della Campania ‘L. Vanvitelli’ c/o Monaldi Hospital, Via L. Bianchi, 80131 Napoli, Italy

**Keywords:** Mesothelioma, Oncogenesis

Correction to: *Cell Death & Disease*

10.1038/s41419-020-02940-w published online 14 September 2020

Owing to an error in production, the original version of this Article contained an error in the author affiliations.

Author Francesca Pentimalli was incorrectly associated with Histopathological Unit, IRCCS-Istituto Tumori “Giovanni Paolo II”, Viale Orazio Flacco 65, 70124 Bari, Italy. The author’s actual affiliation is Cell Biology and Biotherapy Unit, Istituto Nazionale Tumori-IRCCS-Fondazione G. Pascale, I-80131 Napoli, Italy.

We apologise for this error, and confirm that it this has now been corrected in both the PDF and HTML versions of the Article.

