# Correction: P53-regulated miR-320a targets PDL1 and is downregulated in malignant mesothelioma

**DOI:** 10.1038/s41419-026-08742-w

**Published:** 2026-06-11

**Authors:** Caterina Costa, Paola Indovina, Eliseo Mattioli, Iris Maria Forte, Carmelina Antonella Iannuzzi, Luca Luzzi, Cristiana Bellan, Simona De Summa, Enrico Bucci, Domenico Di Marzo, Marisa De Feo, Luciano Mutti, Francesca Pentimalli, Antonio Giordano

**Affiliations:** 1 Cell Biology and Biotherapy Unit, Istituto Nazionale Tumori-IRCCS-Fondazione G. Pascale, Napoli, Italy; 2 Sbarro Institute for Cancer Research and Molecular Medicine, Center for Biotechnology, College of Science and Technology, Temple University, Philadelphia, PA USA; 3 Institute for High Performance Computing and Networking, National Research Council of Italy (ICAR-CNR), Naples, Italy; 4 Histopathological Unit, IRCCS-Istituto Tumori “Giovanni Paolo II”, Bari, Italy; 5 Thoracic Surgery Unit, Department of Medicine, Surgery and Neuro Sciences, Diagnostic Imaging, University of Siena, Azienda Ospedaliera Universitaria Senese, Siena, Italy; 6 Department of Medical Biotechnologies, University of Siena, Siena, Italy; 7 Molecular Diagnostics and Pharmacogenetics Unit-IRCCS-Istituto Tumori “Giovanni Paolo II”, Bari, Italy; 8 Department of Cardiothoracic Sciences, Università degli Studi della Campania ‘L. Vanvitelli’ c/o Monaldi Hospital, Napoli, Italy

Correction to: *Cell Death & Disease* 10.1038/s41419-020-02940-w, published online 14 September 2020

Following publication of this article, an external reader identified a duplication in Figure 5A involving the anti p53 Western blot panel. Upon review, the authors confirmed that the same anti p53 image had been inadvertently used to represent two different cell lines.

To rectify the error, the authors retrieved the original experimental data and reconstructed the figure generation workflow to determine the source of the duplication and to identify the correct blot. All supporting materials from this internal review are submitted with this corrigendum.

The duplicated anti p53 panel in Figure 5A has been replaced with the correct MSTO 211H blot, obtained directly from the original acquisition file. The corrected Figure 5A is provided in this correction.

**Incorrect figure 5** 
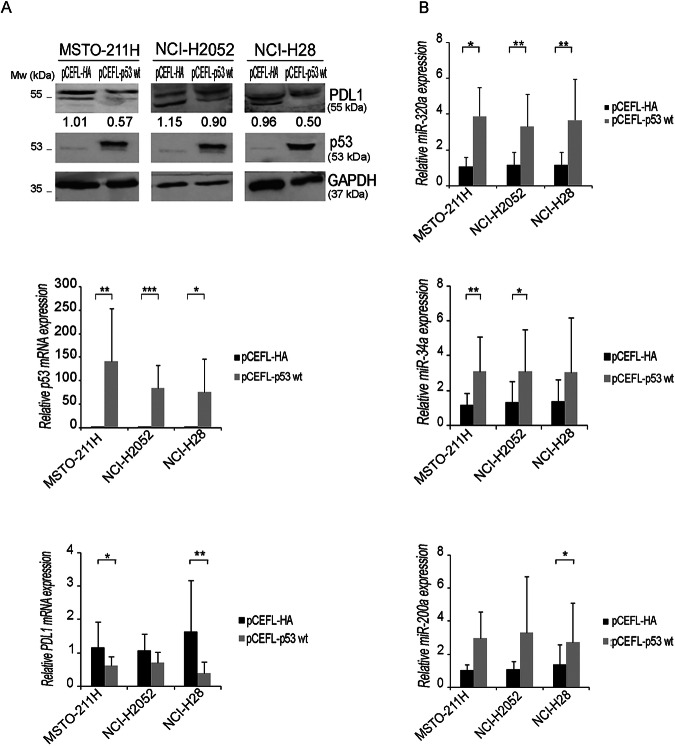


**Correct figure 5** 
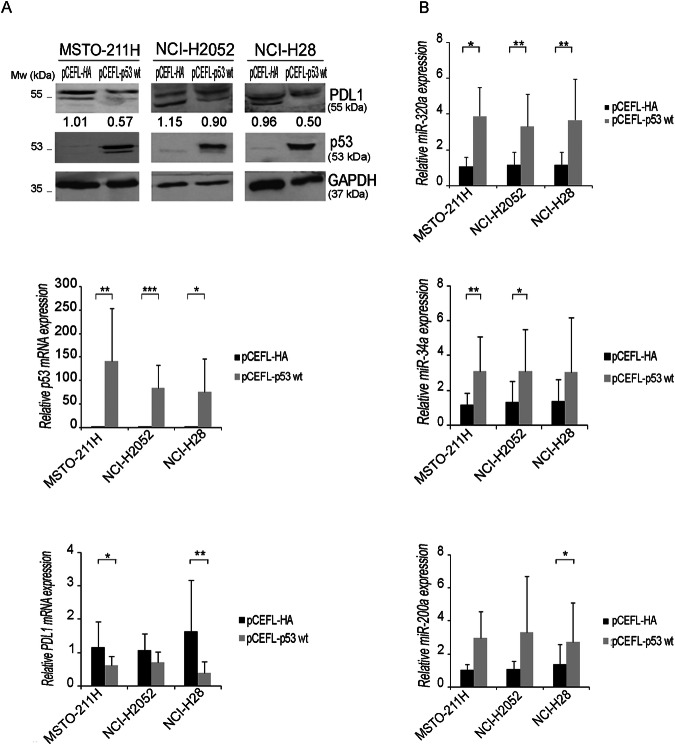


The original article has been corrected.

