# Supplementary figures and images for: P53-regulated miR-320a targets PDL1 and is downregulated in malignant mesothelioma
Source: Cell Death Dis. 2020 Sep 14;11(9):748. doi: 10.1038/s41419-020-02940-w (PMC7490273; doi:10.1038/s41419-020-02940-w)

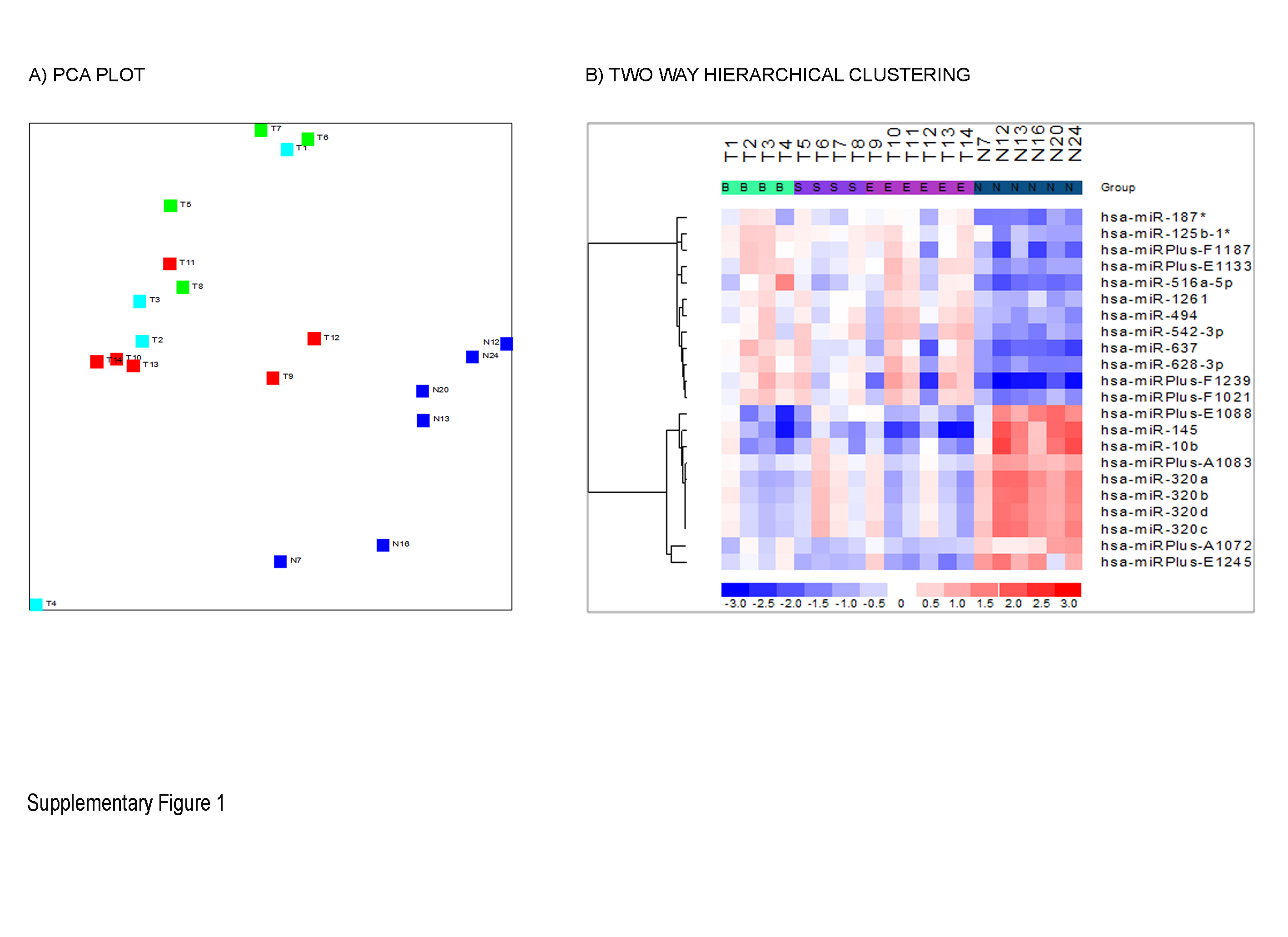

Supplement: Supplementary file 1 — Supplementary Figure 1 [file 41419_2020_2940_MOESM1_ESM.tif]

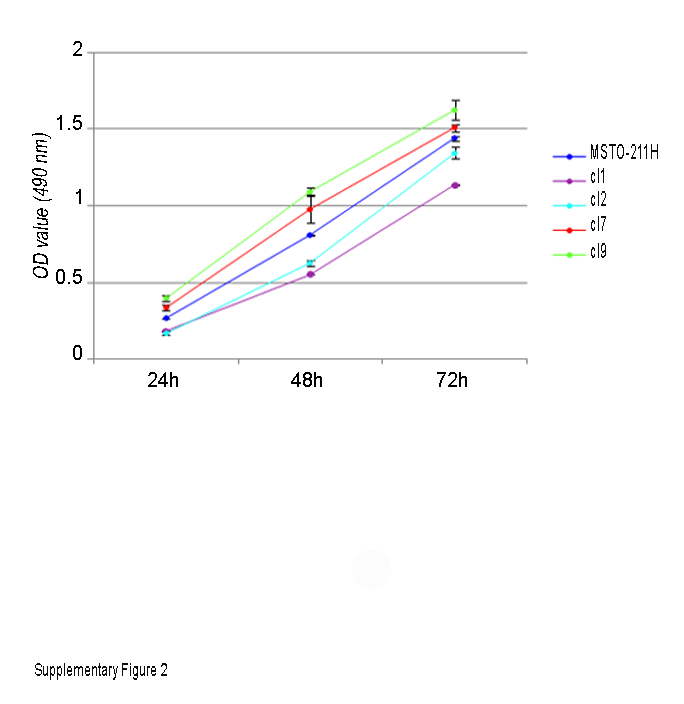

Supplement: Supplementary file 2 — Supplementary Figure 2 [file 41419_2020_2940_MOESM2_ESM.tif]

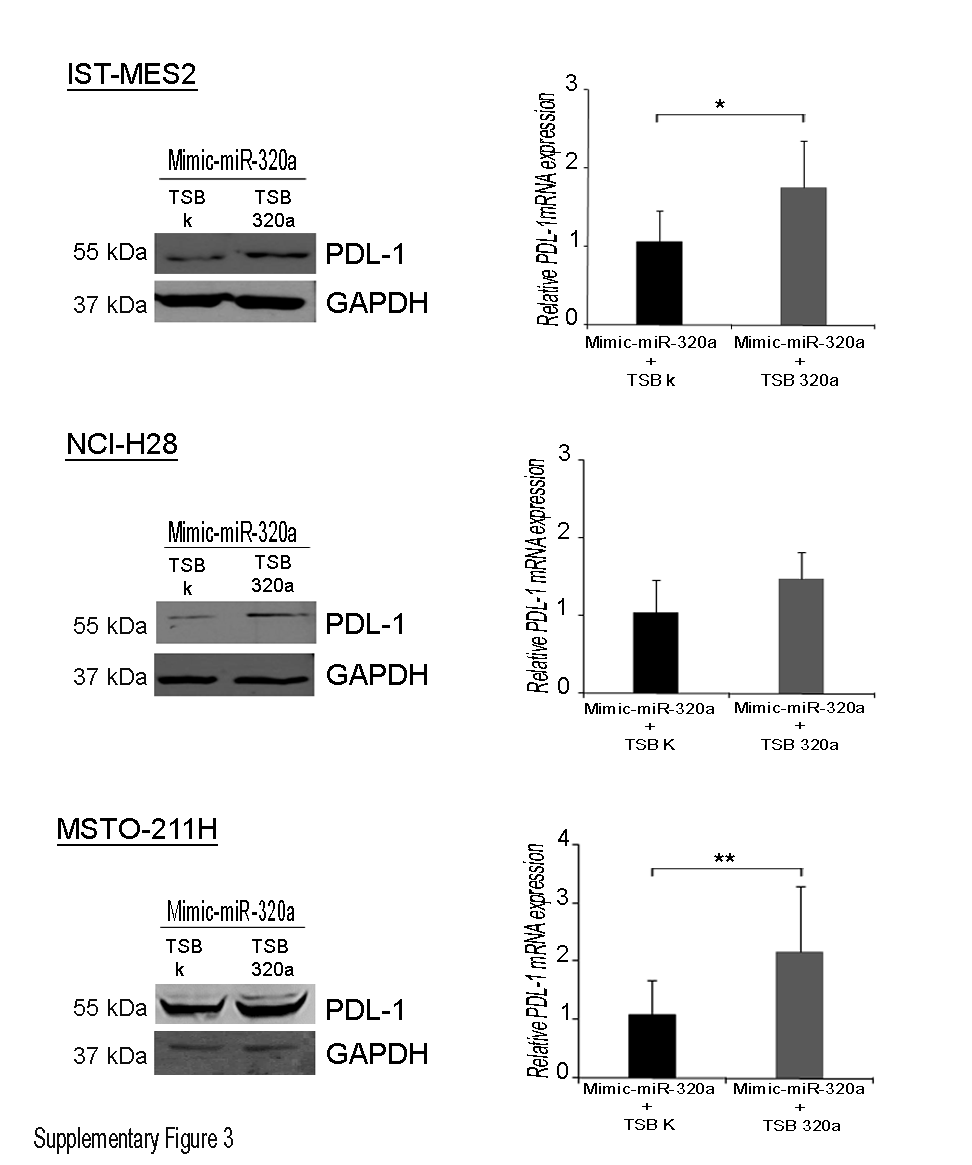

Supplement: Supplementary file 3 — Supplementary Figure 3 [file 41419_2020_2940_MOESM3_ESM.tif]

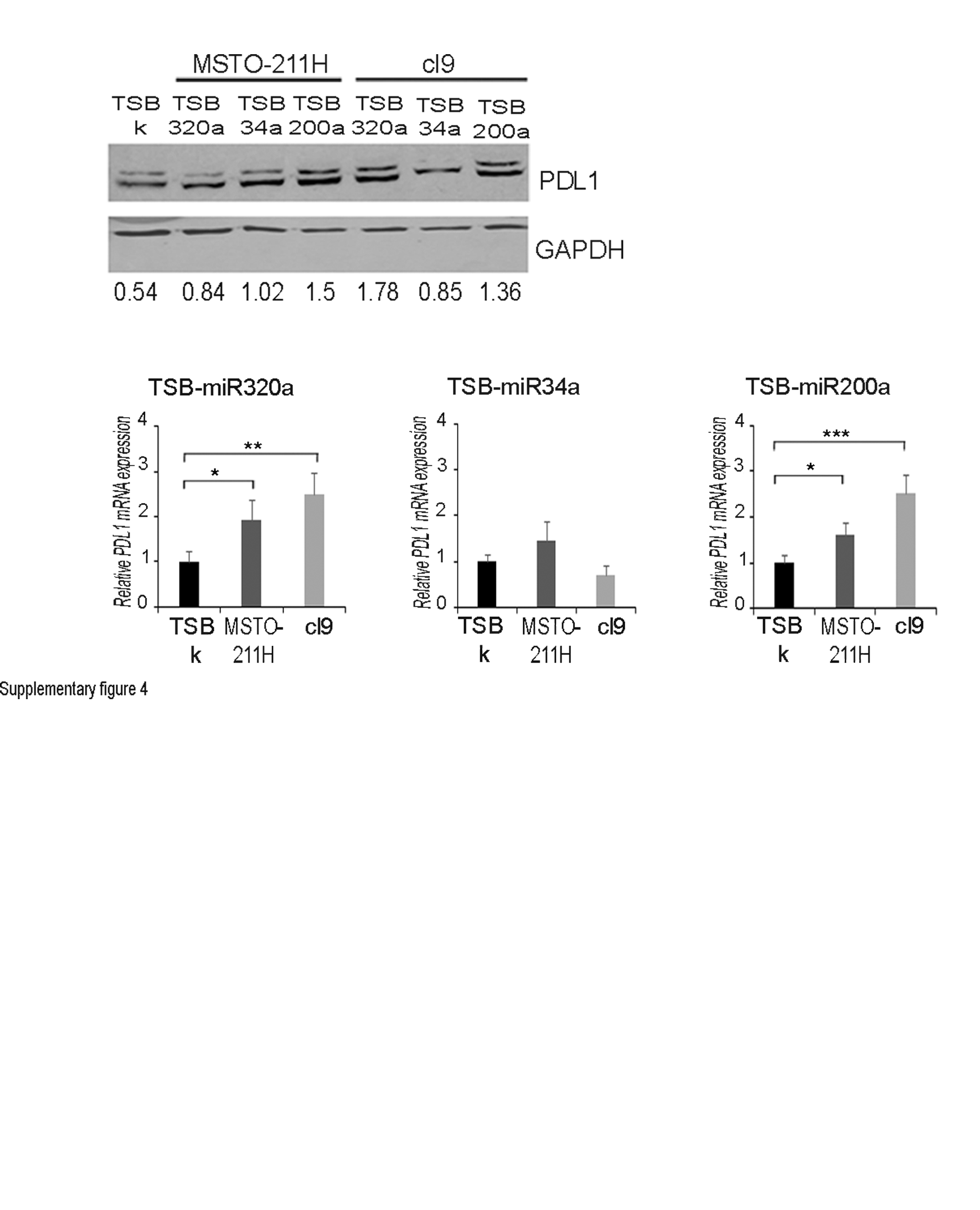

Supplement: Supplementary file 4 — Supplementary figure 4 [file 41419_2020_2940_MOESM4_ESM.tif]
