# Supplementary material for: P53-regulated miR-320a targets PDL1 and is downregulated in malignant mesothelioma
Source: Cell Death Dis. 2020 Sep 14;11(9):748. doi: 10.1038/s41419-020-02940-w (PMC7490273; doi:10.1038/s41419-020-02940-w)
Supplement: Supplementary file 5 — Supplementary Tables [file 41419_2020_2940_MOESM5_ESM.docx]

**Supplementary Table 1 Patient characteristics**

| **Clinical features** | **Screening cohort** | | **Extended series cohort** | | | **Total patients** |
| --- | --- | --- | --- | --- | --- | --- |
| ***Gender*** | | | | | |  |
| **Male** | 12 | | | 15 | | 27 |
| **Female** | 2 | | | 3 | | 5 |
| ***Smoke*** | | | | | |  |
| **Current** | 5 | | | 9 | | 14 |
| **Former** | 1 | | | 1 | | 2 |
| **No smoker** | 7 | | | 2 | | 9 |
| **Unknown** | 1 | | | 6 | | 7 |
| ***Asbestos exposure*** | | | | | |  |
| **Yes** | 10 | | | 10 | | 20 |
| **No** | ― | | | 1 | | 1 |
| **Unknown** | 4 | | | 7 | | 11 |
| ***Stage IMIG*** | | | | | |  |
| **I-II** | 1 | | | ― | | 1 |
| **III** | 9 | | | 11 | | 20 |
| **IV** | 2 | | | ― | | 2 |
| **Unknown** | 2 | | | 7 | | 9 |
| ***Histology*** | | | | | |  |
| **Epithelioid** | 6 | | | 12 | | 18 |
| **Sarcomatoid** | 4 | | | 1 | | 5 |
| **Biphasic** | 4 | | | 5 | | 9 |
| ***Mean age at diagnosis*** |  |  | | | |  |
| **Male** |  | 67.91 | | | 68.46 | 68.22  59.80 |
| **Female** |  | 65.33 | | | 56.33 |  |

**Supplementary Table 2 Primers and target site blockers used in the study**

| **Sequence id** | **Sequence** |
| --- | --- |
| *PDL1*-sense | 5’-GAACGCATTTACTGTCACGGT-3’ |
| *PDL1*-antisense | 5’-TGTTCTTATCCTCCATTTCCCA-3’ |
| *TP53*-sense | 5’-CAGATCCGTGGGCGTGAGC-3’ |
| *TP53*-antisense | 5’-ATGGCGGGAGGTAGACTGAC-3’ |
| β*-actin*-sense | 5’-CAGGGCGTGATGGTGGGC-3’ |
| β*-actin*-antisense | 5’-CTCGGTCAGCAGCACGG-3’ |
| *Mutant PDL1* 3’-UTR mutagenesis forward | 5’-AAACTAGCGGCCGCTAGTTTCCCATA*CG*TTTT-3’ |
| *Mutant PDL1* 3’-UTR mutagenesis reverse | 5’-CTAGAAAA*CG*TATGGGAAACTAGCGGCCGCTAGTTT-3’ |
| miR-320a pmRi-ZsGreen1 forward | 5’-CCGGCGGATTCGACTGGGCCACAGTATTTATC-3’ |
| miR-320a pmRi-ZsGreen1 reverse | 5’-CATTAAAGCTTGACCCCCGAGCGCCGCAG-3’ |
| miR-320a target site blocker | 5’-TAAGTTATCTTTCCCATAGCTTTTCATTATCTTTCATA-3’ |
| miR-34a target site blocker | 5’-TTCCAGAAGCAACTGCTACTGCCTTTCATTCATATGTT-3’ |
| miR-200a target site blocker | 5’-TGCAAAATCACATTTTCTTTCTGGAAATTCCGGCAGTGTA-3’ |

**Supplementary Table 3 MiRPlus sequences**

| **miRPlus** | **Mature sequence** |
| --- | --- |
| hsa-miRPlus-E1245 | GAGGAAGGUGGGGAUGC |
| hsa-miRPlus-F1202 | UGUGGGAGAGGGUAUCAGGGA |
| hsa-miRPlus-E1100 | AGUAAGGUCAGCUAAAUAAGCU |
| hsa-miRPlus-E1065 | AUUGGUCGUGGUUGUAGU |
| hsa-miRPlus-E1108 | AAAAUGUUUAGACGGGCUCAC |
| hsa-miRPlus-E1170 | GUUUAGACGGGCUCACAU |
| hsa-miRPlus-E1172 | AAGAAUGACCGCUGAAGAACGU |
| hsa-miRPlus-E1088 | UGCAGAGUGGGGUUUUGCAGUCCUU |
| hsa-miRPlus-F1187 | AGAGUCGAGAGUGGGAGAA |
| hsa-miRPlus-F1239 | UGGACUGGACUACAAUGAC |
| hsa-miRPlus-F1021 | GGGAGGACAAAGGACUGGC |
| hsa-miRPlus-E1153 | AUGAGGUGGCAAGAAAUGGGCU |
| hsa-miRPlus-E1078 | GACUCUUAGCGGUGGAUC |
| hsa-miRPlus-E1038 | GCAUGAGUGGUUCAGUGGU |
| hsa-miRPlus-A1083 | AAAAGCUGAGUUGAGAGGG |
| hsa-miRPlus-A1072 | CAGAGAGGACCACUAUGGCGGG |

Footnote: a sequence search in miRBase (release 22) identified partial match with the following annotated miRNAs:

hsa-miRPlus-F1202 → hsa-miR-7106-3p

hsa-miRPlus-E1088→ hsa-miR-8077

hsa-miRPlus-F1021→ hsa-miR-3919

hsa-miRPlus-E1153→ hsa-miR-4730
