# Supplementary material for: P53-regulated miR-320a targets PDL1 and is downregulated in malignant mesothelioma
Source: Cell Death Dis. 2020 Sep 14;11(9):748. doi: 10.1038/s41419-020-02940-w (PMC7490273; doi:10.1038/s41419-020-02940-w)
Supplement: Supplementary file 6 — Supplementary Figure legends [file 41419_2020_2940_MOESM6_ESM.docx]

**Supplementary Figure 1 l:** **MiRNA signature in malignant pleural mesothelioma and normal mesothelium specimens. a)** Principal Component Analysis (PCA) was used as a method to reduce the dimensionality of the large dataset. PCA allows to capture the variance in a dataset in terms of principle components and the derived PCA plot provides a way to visualise to which extent the different histological specimens group together. The PCA-plot was performed on log2(Hy3/Hy5) ratios which passed the filtering criteria on variation across samples; LMR>1.5 was selected for comparison to the group "normal". The color code indicates: blue: normal; turqouise: biphasic; green: sarcomatoid; red: epithelioid. **b)** A two-tailed T-test calculated between the tumor versus normal groups identified 22 of 1253 miRNA with p-values lower than 0.0001. The heat map diagram shows the result of the two-way hierarchical clustering of these 22 miRNAs and samples. Each row represents a miRNA and each column represents a sample. The miRNA clustering tree is shown on the left whereas the sample clustering tree is shown at the top. The color scale shown at the bottom illustrates the relative expression level of a miRNA across all samples: a fold change > 1 (red) indicates up-regulation in tumor samples and a fold change < 1 (blue) indicates down-regulation in tumor samples compared to normal samples. Mir-plus sequences and their correspondence to recently annotated miRNAs are reported in Supplementary table 3.

**Supplementary Figure 2 |** **The impact of stable miR-320a modulation on MPM cell viability.** Cell viability of stable MSTO-211H clones, either silenced (cl1 and cl2) or over-expressing (cl7 and cl9) miR-320a, was evaluated through a MTS assay and compared with that of MSTO-211H parental cells (control). The graph reports the means ± SD from two independent experiments. The curves resulted significantly different as evaluated by two-way ANOVA, p<0.0001.

**Supplementary Figure 3 | TSB-320a antagonizes the effect of miR-320a mimic on PDL1 expression levels.** IST-MES2, NCI-H28 and MSTO-211H were transiently co-transfected with mimic-miR320a and 50 nM of either TSB-320a or TSB-K, as a control. Forty-eight hours upon transfection, PDL1 levels were analyzed by western blotting analysis (shown on the left) and real-time qRT-PCR (shown on the right). A representative blot of the protein level is shown. The intensity of the PDL1 bands has been quantified by densitometric analysis using GAPDH as normalizer. Histograms report *PDL1* mRNA relative expression (mean ± SD), n=3 independent experiments. Statistically significant differences were evaluated by paired two-sided Student t-test (* significant, p<0.05; ** very significant, *p*< 0.01).

**Supplementary figure 4 | MiRNA contribution to PDL1 expression in MPM**. MSTO-211H were transfected with TSBs for miR-320a, miR-34a, miR-200a and the corresponding negative control (TSB K). The effect of each TSB was also evaluated in the miR-320a over-expressing cl9 cells. Forty-eight hours later, western blotting analysis was performed using anti-PDL1 and anti-GAPDH antibodies. A representative blot is shown out of three independent experiments. The intensity of the bands was quantified by densitometric analysis and PDL1 band densities were normalized against GAPDH. *PDL1* mRNA levels were determined via real-time qRT-PCR assay at the indicated conditions. Results from three independent experiments are shown. Statistically significant differences were evaluated by one-way ANOVA with Dunnet post-test, to compare all data versus control (* significant, *p*< 0.05; ** very significant, *p*<0.01; *** extremely significant, *p*<0.001). Error bars indicate SD.
